# Open data on health-related neighbourhood features in Great Britain

**DOI:** 10.1038/s41597-019-0114-6

**Published:** 2019-07-01

**Authors:** Konstantinos Daras, Mark A. Green, Alec Davies, Benjamin Barr, Alex Singleton

**Affiliations:** 1 Department of Geography and Planning, University of Liverpool, Liverpool, UK; 2 Institute of Psychology Health and Society, University of Liverpool, Liverpool, UK

**Keywords:** Geography, Social sciences

## Abstract

Our study details the creation of a series of national open source low-level geographical measures of accessibility to health-related features for Great Britain. We create 14 measures across three domains: retail environment (fast food outlets, gambling outlets, pubs/bars/nightclubs, off-licences, tobacconists), health services (General Practitioners, pharmacies, dentists, hospitals, leisure centres) and the physical environment (green space and air quality). Using the network analysis process of Routino, postcode accessibility (km) to each of these features were calculated for the whole of Great Britain. An average score for each domain was calculated and subsequently combined to form an overall Index highlighting ‘Access to Healthy Assets and Hazards’. We find the most accessible healthy areas are concentrated in the periphery of the urban cores, whilst the least accessible healthy areas are located in the urban cores and the rural areas. The open data resource is important for researchers and policy makers alike with an interest in measuring the role of spatial features on health.

## Abstract (summary)

**Table:** 

Design Type(s)	data integration objective • network analysis objective • population data analysis objective
Measurement Type(s)	Health
Technology Type(s)	digital curation
Factor Type(s)	
Sample Characteristic(s)	Great Britain • anthropogenic habitat

Machine-accessible metadata file describing the reported data (ISA-Tab format)

## Background & Summary

The trends of geographical variations in health have long been studied by the research community. In 1842, a representative example of geographical inequality between occupational groups in England was presented by the social reformer Edwin Chadwick. He highlighted that male life expectancy of labourers in Rutland (38) was higher than that of professional tradesmen in Liverpool (35)^1^. The differences that Chadwick observed were due to urban-rural disparities^2^, such as the poor living conditions and air pollution in the urban areas. Almost 175 years after Chadwick’s report wide geographical inequalities are still observed between these regions^3^. Although, today, overall living conditions have improved, there are longstanding spatial differences in the environments we live in that still impact upon our health.

We focus on three main categories of geographical determinants of health. (1) access to retail outlets, (2) access to health services and (3) environmental quality.

1. Accessibility to retail outlets refers to the distance to stores that sell goods or services that, in our interest, may have an impact on health. Specific types of outlets that have shown consistent evidence across the literature include: Access to fast food outlets is associated with obesity^4^; Pubs/bars/nightclubs and off-licences locations are linked to patterns in alcohol-related harms^5^; Gambling outlets density is associated with problem gambling behaviours that are linked to poorer mental wellbeing^6^; Tobacco outlets have been shown to influence smoking patterns^7^.
2. Access to health services (e.g. General Practitioners, Pharmacies, Dentists, and Hospitals) is important since research has shown that individuals who live further away may be less likely to use a service^8^. We also include access to leisure services alongside them since they can also influence positive behaviours such as physical activity^9^.
3. Finally, features of the physical environment have also been shown to influence health. Pollution is a major cause of ill health and is estimated to be responsible for 16% of all deaths globally in 2015^10^. Access to green space, defined as areas of natural vegetation including grasslands and woodlands, has also been associated with lower mortality rates^11^ and improved wellbeing^12^.

To assess the extent that geographical context still matters for explaining spatial patterns in health outcomes, it is important to have data measuring the location of environmental features hypothesised to impact health. However, there are several issues which limit our ability to effectively assess the contribution of these environmental features. Firstly, processing data at high spatial resolutions requires heavy data manipulation. Researchers and policy officials often don’t have the expertise available to them to readily process such data. Secondly, accessibility to these data can be restricted and often consumer data on retail outlets are either not available or privately owned. Finally, where these previous issues have been overcome, data are often not available for all locations at a small spatial scale. Most of studies that have explored the role of these environmental features have been undertaken in local contexts that may not be generalizable to the national level. Where they are available at the national level, this is often only for large geographical zones, which are not always useful.

Our project aims to develop health related geographic indicators at high spatial resolution for Great Britain to minimise the barriers that researchers and policy makers face in investigating the spatial and environmental determinants of health^13^. We outline the process for how each health-related indicator was developed and the creation of a new descriptive tool – the index of ‘Access to Healthy Assets and Hazards’ (AHAH). The indicator takes each measure we have created to generate a summary statistic of the health related accessibility and environmental characteristics of an area. The strength of our index lies in moving beyond simply examining each indicator alone, towards developing a composite measure of neighbourhood quality.

## Methods

**Fig. 1:**
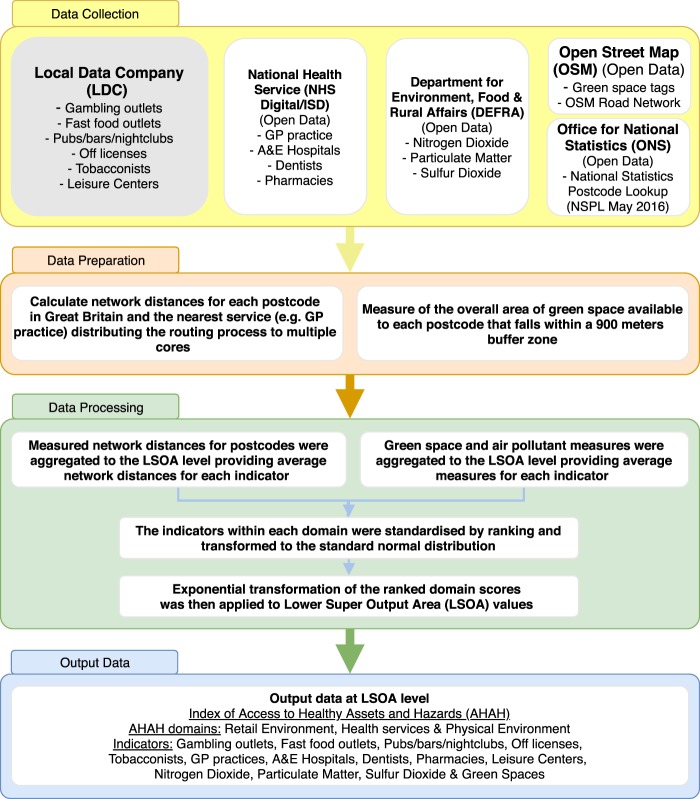
Schematic overview of the data processing method adopted to generate the index and indicators of AHAH.

The AHAH dataset creation involves collecting and processing a large amount of data from several sources in Great Britain. Figure 1 shows the schematic overview of the data processing approach adopted to produce the AHAH index, domains and each of our indicators at Lower Super Output Areas (LSOA) level. Further details of each process are provided in the following sections of the paper.

### Data collection

The AHAH indicators were divided into three main domains: retail environment, health services, and physical environment. For retail environment, data on roughly half a million retail businesses throughout Great Britain were provided by the Local Data Company (LDC) via the Consumer Data Research Centre (CDRC, https://data.cdrc.ac.uk/product/local-data-company-retail-data). The LDC dataset includes the location of business and a hierarchical classification of the type of retail business (39 categories and 370 subcategories). We used this dataset as it is regularly updated through validation via LDC field workers and therefore was more accurate compared to other administrative sources (e.g. Ordnance Survey’s Points of Interest dataset). The LDC operates a 6-month cycle survey to keep this database up to date (about 64000 records per month). Table 1 presents the retail categories selected for inclusion and their prevalence in the dataset.

**Table 1:** LDC categories and subcategories selected for each indicator of the Retail environment domain and the Leisure centres indicator.

Indicator	LDC Category/Subcategory	Business Addresses
Accessibility to Fast food outlets	Chinese Fast Food Takeaway	2,855
	Fast Food Delivery	1,049
	Fast Food Takeaway	11,115
	Fish & Chip Shops	3,829
	Indian Takeaway	1,256
	Pizza Takeaway	2,835
	Sandwich Delivery Service	342
	Take Away Food Shops	8,449
Accessibility to Gambling outlets	Casino Clubs	156
	Bookmakers	8,379
Accessibility to Off-licenses	Off Licences	2,770
Accessibility to Tobacconists	Tobacconists	1,948
Accessibility to Pubs, bars and nightclubs	Night Clubs	1,172
	Bars	4,520
	Public Houses & Inns	18,775
Accessibility to Leisure centres	Leisure Centres & Swimming Baths	727
	Health Clubs	2,738

For the purpose of creating the health services domain, we acquired openly available data from multiple sources. Information was collected on the location of health services (GP practices, hospitals with A&E departments, pharmacies and dentists) from the ODS Access Database of NHS Digital (England and Wales); and the Information Services Division (ISD) in NHS Scotland. Only hospitals with A&E departments were selected as opposed to all hospitals to exclude specialist hospitals that only treat patients on referral and do not provide minor and major trauma care. These data were supplemented with the location of leisure sport centres from the LDC data (see Table 1).

To measure aspects of the physical environment for the associated domain, we integrated two data sources related to air quality and ‘green’ spaces available to public. As a measure of air quality, we used data estimates from Department for Environment, Food and Rural Affairs (DEFRA) for a series of air pollutants that can harm the human respiratory system (NO_2_, PM_10_ and SO_2_)^14^. The air pollution data are model estimates that have been derived from data collected through monitoring sites and estimated levels based on the location of industrial facilities and transport networks. The implementation of these estimates have been modelled under DEFRA’s ‘Modelling of Ambient Air Quality’ contract to provide policy support for DEFRA and are created at a 1 × 1 km resolution^15^. These annual modelled estimates are used to deliver the UK’s reporting obligations to Europe calibrated using data from the Automatic Urban and Rural Network (AURN) for 2015. Additionally, we collected information on public accessible ‘green’ spaces provided by the Geofabrik’s free download web service (http://download.geofabrik.de). The Geofabrik web service has data extracts of countries from the OpenStreetMap (OSM) project which are updated daily. The OSM is ‘volunteered geographical information’ that is contributed by multiple individuals and for Great Britain having comparable quality to other non-open spatial data^16^. From all the available types of ‘green’ spaces in the OSM data, we selected only areas tagged as public accessible with the following area types: cemetery, common, dog park, scrub, fell, forest, garden, greenfield, golf course, grass, grassland, heath, meadow, nature reserve, orchard, park, pitch, recreation ground, village green, vineyard and wood. It is worth noting that even though the golf courses tend to be private lands, some of them include public pathways (e.g. North Shore golf club, Skegness). Therefore, we have included the golf courses with public access into the green space indicator for capturing possible active or passive benefits to the public.

All the input data were collected at the finest level of spatial detail available and as close as possible to 2016. Table 2 lists each variable with additional information about the source and the type of each dataset.

**Table 2:** Input datasets, used to produce the AHAH index and its components.

Name	Source	Publication Date	Data Type	Spatial Resolution
Fast food outlets	Local Data Company (via Consumer Data Research Centre services)	2016	Point	Postcode
Gambling outlets	Local Data Company (via Consumer Data Research Centre services)	2016	Point	Postcode
Off-licenses	Local Data Company (via Consumer Data Research Centre services)	2016	Point	Postcode
Tobacconists	Local Data Company (via Consumer Data Research Centre services)	2016	Point	Postcode
Pubs, bars and nightclubs	Local Data Company (via Consumer Data Research Centre services)	2016	Point	Postcode
GP practices	England & Wales: NHS digital, Scotland: NHS/ Information Services Division (ISD) in NHS Scotland	19^th^ Oct 2016, 1^st^ Jul 2016	Point	Postcode
A&E hospitals	England: NHS digital, Wales: NHS Wales, Scotland: NHS/ Information Services Division (ISD) in NHS Scotland	2014, Feb 2017, Feb 2017	Point	Postcode
Pharmacies	England: NHS digital, Wales: NHS Wales, Scotland: NHS/ Information Services Division (ISD) in NHS Scotland	19^th^ Oct 2016, Oct 2016, 15^th^ Jun 2016	Point	Postcode
Dentist practices	England & Wales: NHS digital, Scotland: NHS/ Information Services Division (ISD) in NHS Scotland	Aug 2016, Oct 2016	Point	Postcode
Leisure services	Local Data Company (via Consumer Data Research Centre services)	2016	Point	Postcode
Green spaces	Open Street Map Foundation & Contributors	Nov 2016	Polygon	Volunteered Geographic Information (VGI) accuracy^16^
Nitrogen Dioxide (NO_2_)	Department for Environment, Food and Rural Affairs (DEFRA)	2015	Raster	1 × 1 km cell
PM10 Particles	Department for Environment, Food and Rural Affairs (DEFRA)	2015	Raster	1 × 1 km cell
Sulphur Dioxide (SO_2_)	Department for Environment, Food and Rural Affairs (DEFRA)	2015	Raster	1 × 1 km cell
Transportation Network	Open Street Map Foundation & Contributors	Nov 2016	Polyline	Volunteered Geographic Information (VGI) accuracy^16^
NSPL lookup table	Office of National Statistics	May 2016	Point	Postcode
Lower Super Output Area/Data Zone boundaries	UK Data Service	2011	Polygon	Lower Super Output Area/Data Zone

### Data preparation

Accessibility measures were derived for each of the AHAH indicators related to service locations (postcodes) and were created using the Routino open source software (https://www.routino.org). Routino is an application for identifying the shortest path between two locations using the OSM transport network and considers directional restrictions on roads as well as attached labels of speed limits and barriers. We measured the network distance between the population-weighted centroid of each postcode in the National Statistics Postcode Lookup (NSPL) and the coordinates of the nearest service (e.g. a population-weighted centroid of postcode for off-license). The NSPL is a database containing all postcodes for Great Britain based on the Ordnance Survey grid reference system to 1 metre resolution. The overall process for calculating about 2 million network distances for each postcode in Great Britain was CPU-intensive because of the sequential computation of distances used by the Routino algorithm. To make use of all the available CPU power (multiple cores), we implemented a parallelisation framework using multiple Docker containers (https://www.docker.com) that run Routino instances in parallel for subsets of 200,000 GB postcodes.

Measured network distances for each indicator were aggregated from postcode into an aggregate geography by taking the average of network distances across the postcodes (mean value). The selected geographies for England and Wales were Lower Super Output Area (LSOA), and for Scotland the Data Zones (DZ). These geographies are relatively small zones which are regularly used in research and local government and are nested within larger statistical geographies. The mean population size of the LSOA areas is about 1,500 people with a minimum of 1,000 and maximum of 3,000 people per LSOA, while the DZ areas are slightly smaller with population sizes between 500 and 1,000 people.

The indicators for the physical environment domain do not represent distances, therefore they require a different approach. The green space indicator has been defined as an area measure of access to green space available to each postcode that intersect with a 900 meters buffer zone. The selection of the 900 metres buffer zone was based on the recommendation of the European Environment Agency that each individual should have access to green space within a 15 minutes’ walk from their residence^17^. Different buffer sizes such as 600, 700 and 800 metres were created for sensitivity testing purposes, however the results did not produce hugely different patterns. For the air pollution measures, we calculated the average modelled values (mean value) of the 1 × 1 kilometre grid cells overlapping with each LSOA area.

### Producing AHAH domains and index

Each indicator was individually standardised by ranking LSOAs from best to worst and its direction of association to health was dictated by the literature where there was a clear positive or negative effect (see Table 3). For example, accessibility to fast food outlets were identified as health negating, whereas accessibility to GP practices were seen as health promoting. We flipped the direction of the overall retail environment and the green space measures during the construction of the index, so all measures were on the same scale/directions. Each variable was then transformed to the standard normal distribution by using the Rankit method^18^ which is a rank-based inverse normal transformation. The indicators within each domain were combined with equal weights forming an overall domain score. We chose to equally weight each indicator since there was no clear justification for different weightings, which otherwise would emphasise the relative importance of the composite scores.

**Tablee 3:** Association of each indicator to health used for the AHAH index.

Domain	Indicator	Hypothesised association to health*	
		Low value	High value
Retail Environment	Accessibility to Fast food outlets	−	+
	Accessibility to Gambling outlets	−	+
	Accessibility to Off-licenses	−	+
	Accessibility to Tobacconists	−	+
	Accessibility to Pubs, bars and nightclubs	−	+
Health Services	Accessibility to GP practices	+	−
	Accessibility to A&E hospitals	+	−
	Accessibility to Pharmacies	+	−
	Accessibility to Dentist practices	+	−
	Accessibility to Leisure services	+	−
Physical Environment	Accessibility to Green spaces	−	+
	Nitrogen Dioxide (NO_2_)	+	−
	PM10 Particles	+	−
	Sulphur Dioxide (SO_2_)	+	−

*A positive value means that a value is positively associated to health i.e. health promoting (and vice versa). We have divided into ‘high’ and ‘low’ values to help assess the direction of the variables e.g. a low value (e.g. 0.7 km) for access to gambling outlets means an area is located nearer to one compared to a higher value (e.g. 10 km).

The following step after the calculation of the three domains was to combine them to an overall AHAH index. To achieve this, we decided to follow the methodology adopted in the 2015 English Index of Multiple Deprivation^19^ because of its robustness and ability to reduce *‘cancellation effects’* between domains. We ranked each domain *R* and scaled it to the range [0, 1] where R = 1/N was defined as the most ‘health promoting’ LSOA and R = N/N for the least promoting (*N* is the total number of LSOAs in Great Britain). To minimise the ‘cancellation effects’ in the overall index, we applied an exponential transformation to the ranked domain scores. This way, high levels of accessibility in the health environment domain are not completely cancelled out by low levels of accessibility in the retail environment domain. Worth noting is that the exponential transformation applied in each domain puts greater emphasis on the LSOAs with poor accessibility and so facilitates identification of the neighbourhoods with the worst health promoting aspects. We selected this approach so that our index can identify areas of poor health-related features, which are more likely to be of interest to policy makers. The exponential transformed indicator score *X* is given by:

$$X=-23\,ln(1-R(1-{e}^{-100/23}))$$

where *‘ln’* denotes natural logarithm and *‘e’* the exponential transformation.

Lastly, we have combined the three domains (retail environment, health services and physical environment) using equal weights to form an overall index of ‘Access to Healthy Assets & Hazards’.

### AHAH index mapping illustration

**Fig. 2:**
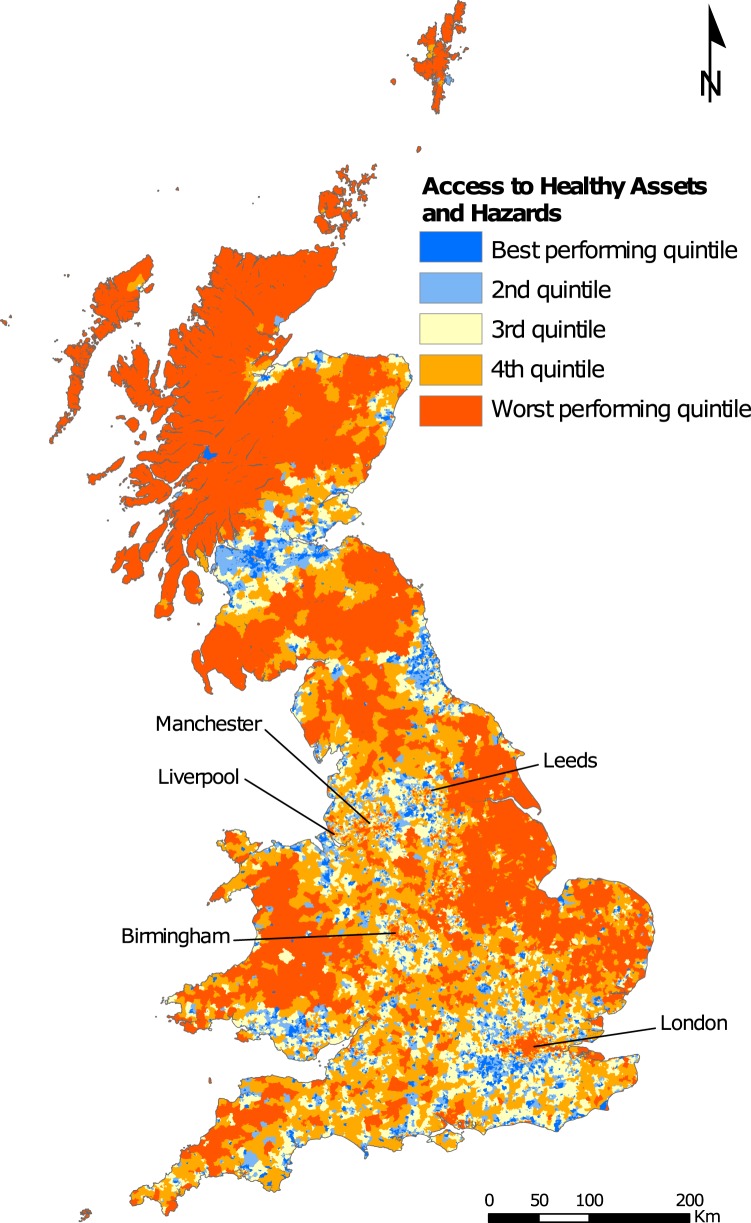
Overall Index of Access to Healthy Assets and Hazards (AHAH) in Great Britain. Best and worst performing neighbourhoods represented with blue and orange colours respectively.

**Fig. 3:**
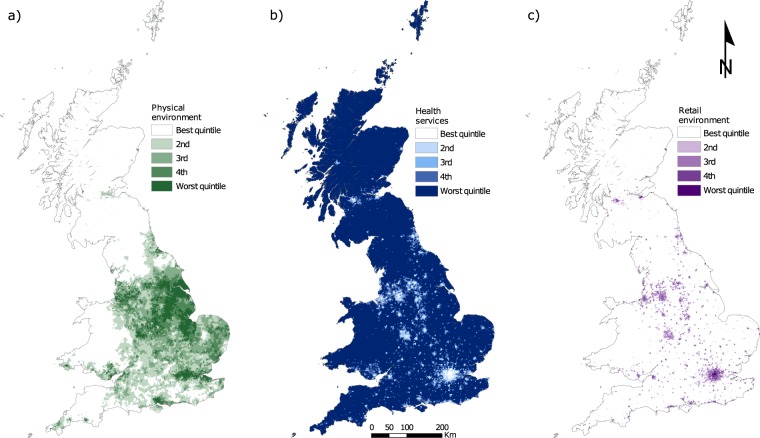
Visualisation of the three domains of the AHAH index in Great Britain. (**a**) Physical environment domain, (**b**) health services domain, and (**c**) retail environment domain.

We mapped the overall index (Fig. 2) and domain scores (Fig. 3) to explore their geographic distributions to contextualise and understand our indicators.

Figure 2 shows the overall index ‘Access to Healthy Assets & Hazards’ (AHAH). The most remote rural areas are identified as ‘unhealthy’ in terms of accessibility in our measure. While they typically performed well on our physical environment and retail domains, they perform poorly on accessibility to health services, due to their remoteness and being sparsely populated. Urban cores of cities such as central London, central Birmingham, and the city centres of areas such as Liverpool, Leeds and Manchester, also perform poorly for our index. These urban centres have high volumes of health services but also have high accessibility to retail related health hazards and higher levels of air pollution. The areas that were identified as the most health promoting through our index are typically smaller towns and suburban areas on the outskirts of cities. This is because these areas were generally located near to health services and green spaces, but further away from polluted environments or retail services that were potentially unhealthy.

The physical environment domain (Fig. 3a) demonstrates better physical environment for rural areas, with higher scores (i.e. unhealthy environments) aligned predominantly to urban areas. The health services domain (Fig. 3b) has a contrasting pattern to that observed for the physical environment. Rural areas have poorer accessibility to health services than urban areas, which is expected due to the distinct differences in infrastructure provision and population density. Plotting quintiles hides some variation between areas particularly in rural areas where remote regions in Wales and Scotland have very poor access to health services. The retail services domain (Fig. 3c) is similar to the health domain, with urban areas again clearly defined. The direction of the relationship has reversed for the retail domain, with urban areas having higher accessibility to health negating features in contrast to health services. Rural areas perform better in comparison with urban areas here, due to being located far away from them.

## Data Records

All the output datasets described in this article, are publicly available through the CDRC website (https://data.cdrc.ac.uk/dataset/access-to-healthy-assets-and-hazards-ahah) under the UK Open Government Licence (OGL). The datasets stored in the CDRC repository (also available within the figshare repository^20^) represent the outputs produced for the latest version of the AHAH index and components (Table 4). Updated versions of AHAH index will become available in the future for including additional components and attaining better quality. An online map of AHAH domains and the overall index for the whole of Great Britain is available through CDRC interactive web mapping tool (https://maps.cdrc.ac.uk/#/indicators/ahah).

**Table 4:** Name, description and data citations of the AHAH datasets as described in this article.

Name	Description	Data citation
1. AHAH Inputs/Components (CSV file)	Contains the measures and deciles of all the index components at the LSOA level in the Great Britain (Accessibility to Fast food outlets, Gambling outlets, Off-licenses, Tobacconists, Pubs, bars and nightclubs, GP practices, A&E hospitals, Pharmacies, Dentist practices, Leisure services, Green spaces, Nitrogen Dioxide (NO2), PM10 Particles, and Sulphur Dioxide (SO2)).	figshare source^20^
2. AHAH Overall Index and Domains (CSV file)	Contains the deciles and scores of AHAH index and the three domains at the LSOA level in the Great Britain (Retail environment, Health services and Physical environment).	figshare source^20^

## Technical Validation

Datasets produced by this paper have been obtained by processing input source data to produce open data outputs of both the AHAH domains and the overall index at the LSOA level. The source data related to health services and air pollution are already validated by the NHS digital and DEFRA respectively. The retail location data are assembled and validated on a rolling basis by LDC company and are collected through field researchers visiting approximately 70,000 stores monthly. The OSM transport network and the OSM green spaces are crowdsourced datasets that are contributed by multiple individuals and for Great Britain have comparable quality to other non-open spatial data^16,21^. Ground-truth validation was conducted to determine the degree to which the OSM network inaccuracies are affecting the estimated network distances at postcode level for each indicator. We used the Quantum GIS software for manually checking the estimated network distances at postcode level and the aggregated distances at LSOA level against the input data sources for selected urban and rural neighbourhoods. We detected small discrepancies in the network distances, primarily in remote rural areas that could be attributed to the underline quality of the OSM network but none of them had a significant effect on the aggregated distances at LSOA level. An extensive discussion about the data sources, their quality and a sensitivity analysis of our data outputs is also available in the supplementary appendix of the paper by Green *et al*.^13^.

## Usage notes

One strength of a composite indicator like AHAH index is the ability to provide a powerful means of communication to support decision making. It can synthesise complex multidimensional issues into a single aggregated measure that is easy to interpret (i.e. through ranking areas). However, the AHAH index, like all the composite indices, comes with several limitations related to both conceptual and data issues. While the development of AHAH index that incorporates multiple features of our environment is important, it assumes a framework that these features can be separated or projected into a linear scale of values that either promote or negate health within a neighbourhood. Measures such as off-licenses and tobacconists will underestimate the issues they intend to measure, since alcohol and tobacco can be purchased wider than purely specialist outlets.

The input measures included in AHAH do not constitute all features of environments that may influence health. We focused on environmental aspects where the direction of association to health was clear (i.e. only positive or negative), was supported throughout the literature with consistent evidence demonstrating an association, and for which data were available to measure. There are clearly other determinants of health that will potentially have are greater impact on the spatial distribution of health, such as housing quality, access to jobs, income and access to quality education. The use of the AHAH index in explaining these patterns in health will need to take these into account.

The measures in the AHAH index largely capture distance to facilities that may influence health, they do not capture the size of the provision at those facilities, e.g the number of doctors in a GP practice, or the nature of provision at those facilities – e.g the quality of healthcare provided by a GP practice. Both of these factors will influence the extent to which distance is likely to influence health. We aim to continue to expand the suite of indicators in future years to further broaden our resource.

Another limitation is that we weighted each indicator equally in the overall domain scores knowing that they may not contribute equally towards influencing health. Ideally, we would weight each indicator based on its causal relationship to health, however there is insufficient evidence from the literature to identify these weights. Identifying the relative contributions of each domain and input would not only be useful both to refine AHAH to accurately reflect healthy environments but would also aid policy makers to prioritise which aspects of environments to tackle. Through making all the inputs openly available we hope to enable research into their impact on health, additionally this allows users to alter and refine how the index is constructed to reflect their needs.

Also, we investigated the correlation between the indicators that form our three domains before we apply any transformation on them. There was moderate to strong positive correlation between each of the accessibility measures suggesting they are capturing similar processes related to the urban structure and the co-clustering of services. There was also moderate correlation between the three air quality metrics demonstrating their similarity in values and they were each negatively correlated to all the other measures. The green space measure displayed no association to any other variable, and this was expected because it measures a different type of accessibility defined as area size. Looking at the associations between the domains and overall index scores, the health domain has had little association to the overall score, whereas the other two domains were positive correlated to the overall score.

Two-thirds of the AHAH index consists of accessibility measures (network distances), it is important to highlight the relative nature of these measures. Travelling a distance of 10–15 kilometres to access a pub might be reasonable in rural areas but is unusual in an urban area. It demonstrates the need to extend our indicators to account for the locational contexts to understand areas of poor or good health-related features. Furthermore, in the green space measure, we followed the recommendation of the European Environment Agency that each person should have access to green space no further than 900 metres (or a 15 min walk) from their residence. Such a definition is also open to debate and may vary within different contexts as well. Developing context specific indicators might offer one opportunity for future research. We also selected all green spaces that were accessible to the public within this definition to cover all types that individuals may benefit from actively or passively. We acknowledge that such an approach could be refined by identifying which types of green space people benefit from and how/why, to improve our general measure.

In summary, the AHAH input measures and the overall index offer a useful open resource for understanding the accessibility of neighbourhoods to 14 health-related features of the built and physical environment. We provide one of the most comprehensive sources of small area data within the Health Geography field that is openly available, and our indicators are relevant to policy makers, researchers and the public interested in public health issues. All data including the index, domain scores and input values are available to downloaded freely via the CDRC interactive web mapping tool (the postcode level input measures are also available upon request via the CDRC services).

## ISA-Tab metadata file

Download metadata file

## Data Availability

The program code to produce the AHAH index and components is open source and available through either the GitHub repository(https://github.com/GDSL-UL/AHAH) or figshare repository^22^. The code consists of two R script files containing the commands for building: a) the network distances between each postcode in GB and the nearest service using the Routino tool and the OSM transport network data and b) the scores of AHAH domains and the overall index. Each file is internally documented to explain purpose and, when required, to guide the user in the appropriate script customization. The R scripts, also make use of the *FNN* package^23^ and its k-nearest neighbour search algorithm and the *splitstackshape* package^24^ for reshaping wide data, even when the data are unbalanced.
